# An Ultra-Wide Band MIMO Antenna System with Enhanced Isolation for Microwave Imaging Applications

**DOI:** 10.3390/mi14091732

**Published:** 2023-09-03

**Authors:** Saad Hassan Kiani, Huseyin Serif Savci, Mehr E Munir, Ahmed Sedik, Hala Mostafa

**Affiliations:** 1 Electrical and Electronics Engineering Department, Faculty of Engineering and Natural Sciences, Istanbul Medipol University, 34810 Istanbul, Turkey; 2 Smart Systems Engineering Laboratory, College of Engineering, Prince Sultan University, Riyadh 11586, Saudi Arabia; asedik@psu.edu.sa; 3 Department of the Robotics and Intelligent Machines, Faculty of Artificial Intelligence, Kafr Elsheikh University, Kafr el-Sheikh 33516, Egypt; 4 Department of Information Technology, College of Computer and Information Sciences, Princess Nourah bint Abdulrahman University, P.O. Box 84428, Riyadh 11671, Saudi Arabia

**Keywords:** mmwave, MIMO system, ECC, gain, efficiency, pattern diversity

## Abstract

This paper introduces a novel two-port ultra-wideband (UWB) multiple-input multiple-output (MIMO) antenna system with enhanced isolation characteristics. The antenna, designed on a thin 0.787 mm RO5880 substrate, achieves a compact form factor of 52 × 26 mm${}^{2}$ and offers a wide bandwidth of 9.2 GHz (2.3 GHz to 11.5 GHz) while meeting the VSWR 2:1 criterion. Notably, the proposed antenna demonstrates an impressive increase in isolation, up to 16 dB, through the integration of a shared radiator with small rectangular slots, effectively reducing interference and improving overall performance. Furthermore, a comprehensive analysis of additional MIMO performance parameters, including the envelope correlation coefficient (ECC) and diversity gain, confirms their satisfactory limits, validating the potential of the proposed UWB-MIMO antenna for various UWB applications. The time domain analysis of the UWB antenna is also analyzed, and results are found to be within satisfactory limits. Simulation and measurement results further support the practicality and effectiveness of the antenna design, highlighting its compact size, wide bandwidth, and enhanced isolation characteristics, positioning it as a promising solution for advanced UWB microwave imaging systems.

## 1. Introduction

Microwave imaging antennas require a wide bandwidth, consistent radiation performance, and minimal signal distortion when transmitting pulses through human tissues. Ultra-wideband (UWB) technology, with its short pulse durations, helps achieve these criteria. Compact planar antennas are favored for their small size and integration feasibility in microwave imaging systems. The introduced UWB antennas are highly valuable components in modern communication systems, offering a range of benefits and applications. These antennas operate across an exceptionally wide frequency range, typically spanning several gigahertz [1,2]. Their unique characteristics make them particularly useful in various domains, including wireless communication, radar systems, and sensing applications. One of the key advantages of UWB antennas is their ability to transmit and receive signals over a wide frequency spectrum. This wide bandwidth allows for high data rates and efficient communication. UWB technology enables short-range wireless connectivity with high capacity, making it suitable for applications such as wireless USB, wireless audio and video streaming, and high-speed data transfer [3]. Moreover, UWB antennas are well suited for positioning and tracking systems due to their fine time resolution and accuracy. By utilizing precise time-of-flight measurements, UWB technology enables the accurate localization of objects in both indoor and outdoor environments. This capability has found applications in asset tracking, indoor navigation, and even in healthcare for tracking patients or monitoring vital signs.

When combined with multiple-input multiple-output (MIMO) technology, UWB antennas offer additional benefits and improvements in communication systems [4,5]. MIMO UWB systems utilize multiple antennas at both the transmitting and receiving ends to enhance data throughput, system capacity, and link reliability. By exploiting the spatial domain, MIMO UWB systems can effectively mitigate multipath fading and interference and improve overall signal quality. The benefits of MIMO UWB antennas include increased data rates, extended coverage range, and improved system robustness. By employing multiple antennas, MIMO UWB systems can achieve spatial multiplexing, allowing for the simultaneous transmission of multiple data streams over the same frequency band. This results in higher data rates and increased capacity, meeting the demands of bandwidth-intensive applications [6].

Additionally, MIMO UWB antennas provide an improved coverage range by leveraging spatial diversity. By receiving signals from multiple paths and intelligently combining them, these antennas effectively combat the challenges posed by fading and signal attenuation, ensuring reliable communication even in challenging environments [7]. Furthermore, MIMO UWB systems exhibit better interference rejection capabilities. The presence of multiple antennas allows for advanced signal processing techniques such as beam-forming, which enables the focusing of transmitted signals toward the intended receiver while minimizing interference from other sources. This improves signal quality and enhances the overall system performance. In the literature, several UWB MIMO systems have been proposed [8,9,10,11,12,13,14,15,16]. These UWB MIMO systems comprise different isolation enhancement techniques, including defected ground structure [DGS] [8,9,10], meander line resonators [MLs] [11,12], electromagnetic bandgap structures [EBGs] [13,14], shortening pins [SPs] [15], decoupling structures [DSs] [16], metamaterials, and different configuration techniques [17,18,19,20]. In [10], the authors present a compact (MIMO) antenna system tailored for ultra-wideband (UWB) applications. The physical dimensions of the antenna are notably confined, measuring a mere 22 × 26 mm². The principal objective of the study pertains to the enhancement of isolation within the MIMO antenna configuration. This is accomplished through the strategic integration of two DGSs within the antenna’s immediate vicinity. The first DGS assumes a T-shaped slot geometry etched into the ground plane. This DGS firstly functions as an extender of the antenna’s current path, thereby mitigating the resonant frequency of the antenna at its fundamental mode. Secondly, it acts as a suppressor of surface currents, thereby ameliorating isolation within the frequency band spanning 4 to 10.6 GHz. In addressing another isolation concern, specifically within the frequency range of 3 to 4 GHz, a line slot is introduced within the ground plane. This novel design facilitates a form of controlled coupling that is deliberately configured to offset the undesired coupling that would otherwise adversely impact the antenna’s performance. The line slot functions akin to a neutralization line, effectively counteracting the undesired mutual effects of coupling. A distinguishing aspect of this methodology is its departure from traditional neutralization line techniques, which conventionally introduce additional conductive elements either on the radiation patch or the feeding line to establish supplementary current pathways between antenna elements. In contrast, the proposed DGS approach maintains a diminished influence on the antenna’s intrinsic impedance characteristics, primarily due to the fact that the supplementary current pathways are realized through the judicious etching of coupling slots on the ground plane. This circumvents the need for extensive physical modifications to the antenna structure and preserves its baseline performance attributes. Similarly, in [11], a two-port UWB MIMO configuration is introduced, featuring a floral geometry. This MIMO arrangement shares a common ground plane and incorporates a centrally located I-shaped slot. Each radiating element is adorned with three elliptical patches, resembling flower petals, symmetrically disposed at angular intervals of 60 degrees. The augmented ground structure branches serve a dual purpose. On one hand, they engender multiple resonant frequencies, effectively broadening the system’s frequency bandwidth through resonance mechanisms. On the other hand, these enhanced branches play a pivotal role in securing a heightened level of isolation between the radiating patches. This isolation is achieved by adeptly absorbing the current and thus mitigating mutual coupling effects. The comprehensive dimensions of the MIMO system amount to 30 × 18 mm, while maintaining an impressive error correction coding (ECC) value of less than 0.08.

Reference [12] introduces a four-port multiple-input multiple-output (MIMO) system designed for ultra-wideband (UWB) applications. This system is characterized by distinctive notch filtering attributes and the inclusion of a parasitic isolator. The core of the decoupling strategy comprises a metallic element of square configuration, housing an internal circular slot, accompanied by half-semicircular slots positioned at each corner. To enhance isolation by a margin exceeding 15 dB, four rectangular metallic stubs are ingeniously extended from opposing parallel facets of the structure. Moreover, to cultivate an even greater degree of isolation, the design incorporates rectangular notch features. This is accomplished by integrating two electromagnetic bandgap structures onto the antenna’s reverse side, in conjunction with a shorting pin connecting them to the main radiator element. A UWB MIMO system in [13] incorporated an M-shaped tunning stub to enhance the isolation up to 16 dB. Similarly, the insertion of the stub in [14] reduced coupling up to 14 dB. A decoupling network in a two-port UWB system improved isolation over the entire operating bandwidth up to 14 dB [15]. To enhance isolation, a rectangular loop resonator metamaterial was employed [16] around a radiating element measuring 40 mm × 80 mm. The proposed design achieved an impedance bandwidth of 4.5–8 GHz, isolation below −25 dB, and ECC less than 0.02.

The study introduces a dual-port UWB MIMO system, encompassing a frequency range spanning from 2.3 to 11.5 GHz. To bolster the isolation between the radiating components, a common radiator adorned with compact rectangular slots is employed. The integration of these slots engenders a notable enhancement in isolation levels among the radiating entities. The design is structured as a two-port system, and the computational outcomes are substantiated through empirical measurements. Across various MIMO performance metrics and temporal domain analyses, the outcomes consistently exhibit favorable adherence to anticipated benchmarks.

## 2. Antenna Design

**Figure 1:**
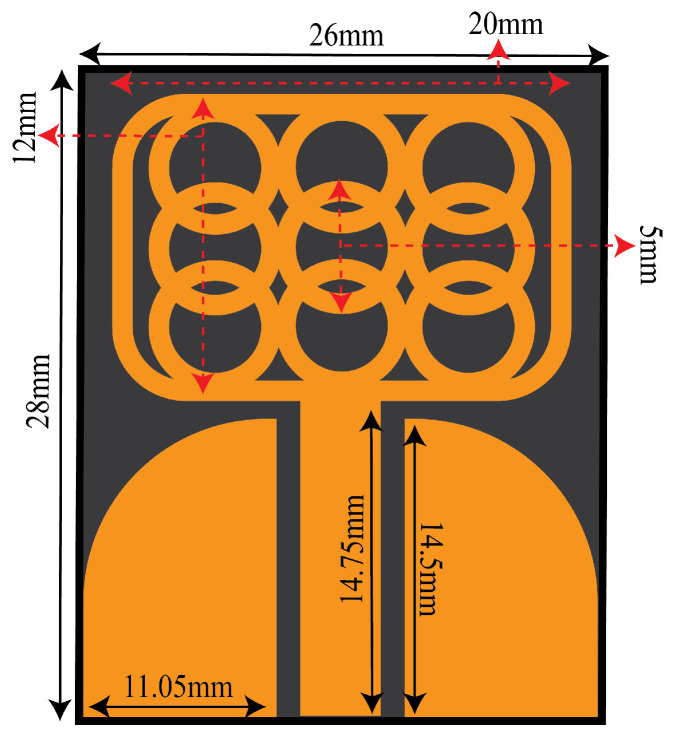
Proposed CPW UWB Antenna.

Figure 1 shows the proposed UWB antenna. The proposed coplanar waveguide antenna is developed using Ro5880 material, which has a relative permittivity of 2.3 and a thickness of 0.787 mm. The CPW feed technique is employed due to its various advantages, such as low losses, coplanar nature, and ease of fabrication.

**Figure 2:**
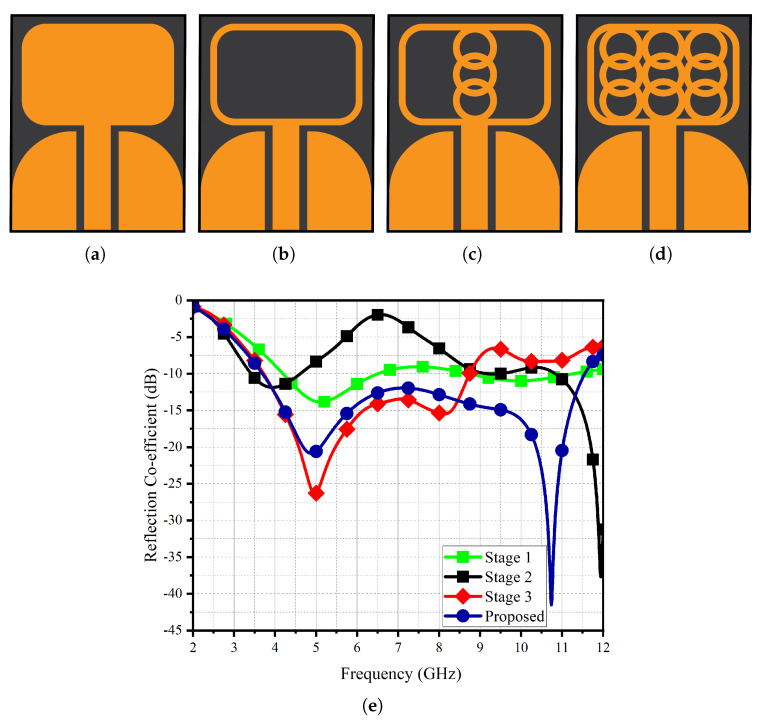
Design Evolution: (**a**) Stage 1, (**b**) Stage 2, (**c**) Stage 3, (**d**) proposed, (**e**) reflection co-efficient.

Figure 2 illustrates the design evolution of the proposed UWB antenna. In the initial stage (Stage 1) of the design process, the architecture consisted of a framework accompanied by a ground plane shaped like a fin. This configuration exhibited a relatively feeble resonance profile spanning from 5 to approximately 7 GHz and 9 to 11 GHz. In Stage 2, the introduction of the framework along with a hollow shell led to a bifurcated resonant behavior. This dual-mode resonance encompassed frequencies within the interval of 3 to 5 GHz, as well as extending beyond 9 GHz. Nonetheless, the reflectance coefficient within the first frequency band exhibited suboptimal performance, with the dominant resonance being primarily observed at 9 GHz and higher frequencies. Subsequent to the integration of circular rings during Stage 3, the resonance phenomenon underwent a downward frequency shift, culminating in the establishment of a broadband trait extending from 3.3 to 8.5 GHz. In the ultimate developmental stage, the quantity of internal circular rings was augmented along each corresponding facet of the framework, resulting in a cumulative nine circular rings nestled within the framework’s confines (as illustrated in Figure 2d). This refined design configuration manifested a frequency response spanning the spectral range from 3.2 to 11.5 GHz.

**Figure 3:**
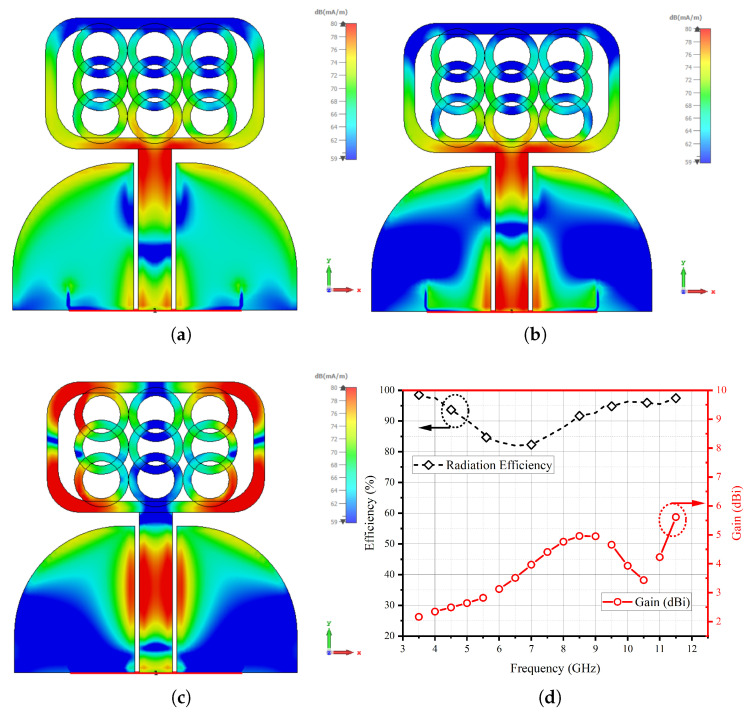

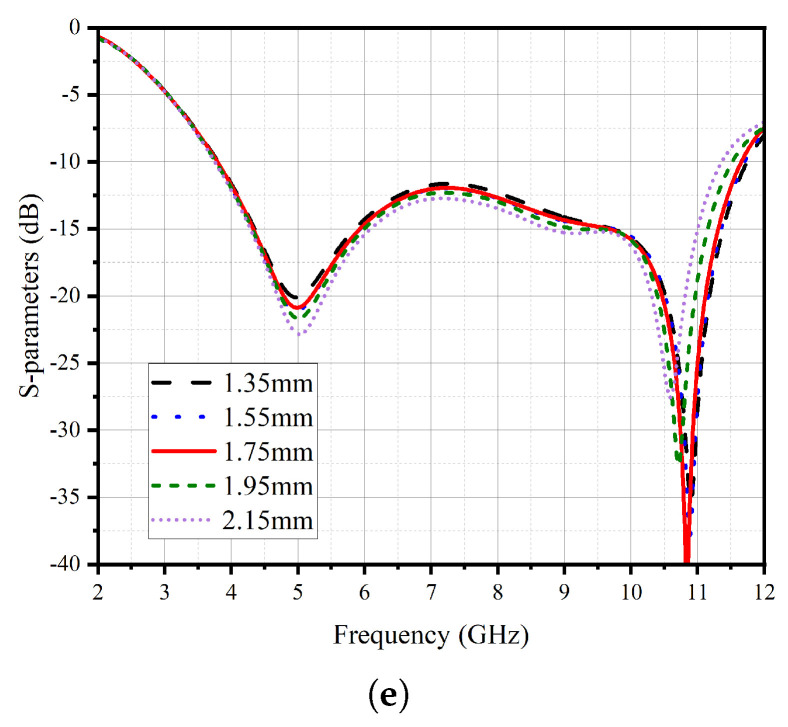
Surface current patterns at (**a**) 4 GHz. (**b**) 7 GHz. (**c**) 10.5 GHz. (**d**) Efficiency and gain. (**e**) Circular ring thickness parametric analysis.

Figure 3 presents a comprehensive analysis of the UWB antenna’s surface current patterns and its performance in terms of efficiency and gain. The intricate details depicted in the figure allow us to discern crucial insights into the antenna’s behavior. Upon careful observation of the surface current patterns, it becomes evident that the circular rings play a pivotal role in generating higher resonance. This is evident from the conspicuous concentration of current intensity observed at the locations of these circular rings. Additionally, the upper rectangular frame also exhibits significant current intensity, further contributing to the overall performance of the antenna. Notably, the efficiency of the UWB antenna has been meticulously evaluated and is found to exceed 85%. High efficiency is a desirable characteristic in antennas, as it indicates the antenna’s ability to effectively convert input power into radiated electromagnetic waves. The distinctiveness of the UWB antennas current distribution can be attributed to its specific design and geometry, which have been engineered to optimize its performance across a wide frequency spectrum. The circular rings and the upper rectangular frame are likely configured to provide enhanced broadband characteristics, enabling the antenna to operate efficiently over a broad range of frequencies. Moreover, the higher resonance generation achieved by concentrating current intensity in the circular rings and upper rectangular frame is likely instrumental in achieving the UWB antenna’s impressive efficiency. The outer ring of the structure is characterized by a radius measuring 2.5 mm, while the inner ring exhibits a radius of 1.75 mm. Evidently, when the inner radius experiences a reduction, a corresponding decrease in the resonance response is observed, primarily noticeable from the terminal frequency point of 11 GHz. Conversely, when augmenting the inner radius and concurrently thinning the circular rings, the resonance response undergoes a broadening phenomenon. It is worth noting that this broadening trend persists until the inner radius reaches the threshold of 1.75 mm. Subsequent to this point, any further adjustments to the inner radius cease to significantly impact the response. As such, the optimal parameter setting is determined to be precisely 1.75 mm. The outcomes of this systematic investigation are visually depicted in Figure 3e for reference.

**Figure 4:**
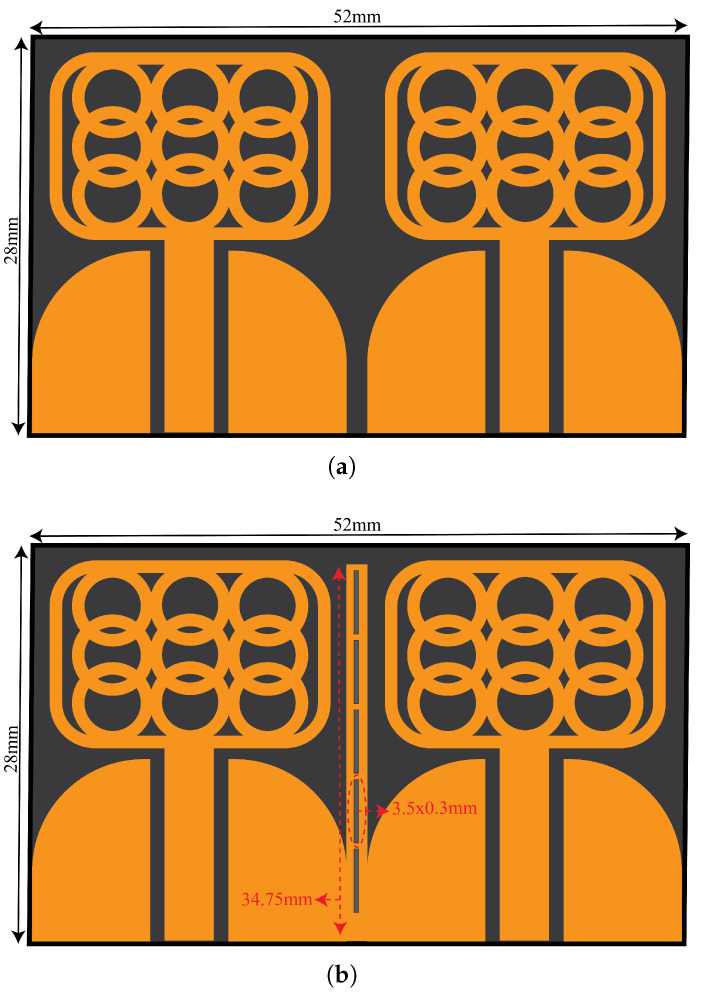
Proposed CPW-fed UWB MIMO Antenna System (**a**) without Isolating Structure. (**b**) With Isolating Structure.

Figure 4 presents the detailed dimensions of the proposed UWB a MIMO configuration with and without isolating structure. The isolating structure comprises a copper strip with five etched slots measuring 3.5 × 0.3 mm. By incorporating the copper strip, the reflection coefficient response of the UWB antenna shifted to lower frequencies of 2.4–10.8 GHz.

**Figure 5:**
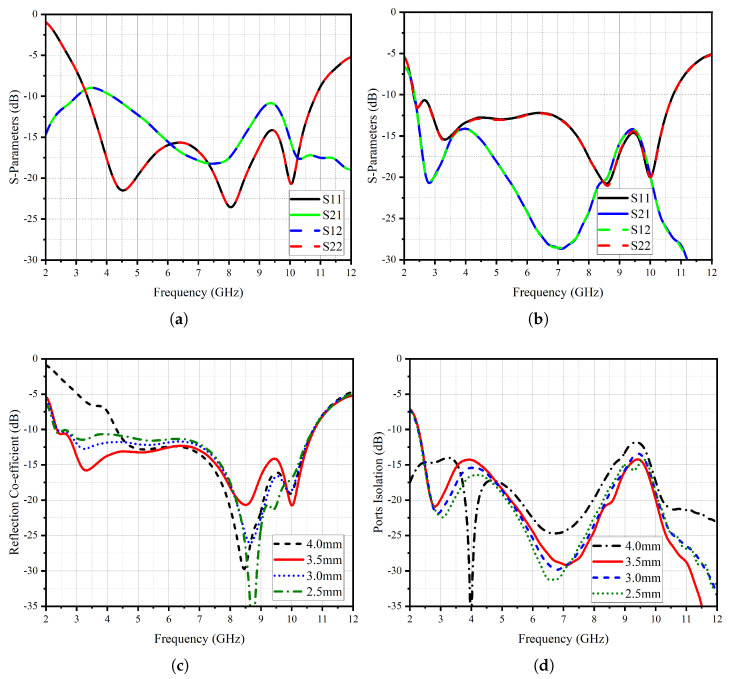
Proposed CPW-fed UWB MIMO Antenna System (**a**) without Isolating Structure and (**b**) with Isolating Structure. (**c**) Reflection Co-efficient with Different Slot Length at Isolating Structure and (**d**) Port Isolation with Different Slot Length at Isolating Structure.

Figure 5 shows an analysis of the S-parameter response of the UWB MIMO system, offering valuable insights into the performance of the antenna array. The results presented in this figure shed light on the reflection coefficient response of the resonating elements and the isolation characteristics, both with and without the implementation of an isolating structure. The reflection coefficient response of the proposed UWB antenna with the insertion of the isolating structure exhibits a remarkable improvement. This enhancement is evidenced by an extended frequency range, where the antenna demonstrates a strong response. Specifically, the lower frequency limit of 2.4 GHz is significantly augmented, reaching an impressive higher response limit of 10.8 GHz. An essential aspect of the UWB MIMO system is its ability to maintain a high degree of isolation between its individual radiating elements.

The figure portrays that in the absence of the isolating structure, the coupling among the radiating elements results in a coupling factor of 10 dB. However, with the strategic introduction of the isolating structure, the S21 and S12 values, representing the level of coupling, experience a substantial enhancement of up to 16 dB across the entire resonating bandwidth. This significant improvement in isolation is a testament to the effectiveness of the proposed isolating structure. The key to achieving such isolation enhancement lies in the incorporation of strategically placed slots within a common radiator. These slots play a crucial role in effectively decoupling the radiating elements, reducing interference, and mitigating the adverse effects of coupling. By intelligently distributing the slots, the isolating structure ensures that each radiating element operates with minimal interaction, leading to improved isolation and enhanced performance of the MIMO antenna system. The UWB MIMO antenna system with the proposed isolating structure not only exhibits an extended frequency range but also achieves significantly improved isolation characteristics. The impact of the slot length within the shared radiator configuration is illustrated in Figure 5c,d, showcasing its influence on both the reflection coefficient and port isolation. Notably, when the slot length is set at 4 mm, a pronounced resonance response is observed, exhibiting a narrowed bandwidth of 6.5 GHz. This bandwidth is confined between the initial frequency of 4.5 GHz and the concluding resonance point of 11 GHz. Concomitantly, at this specific slot length, a minimum isolation level of 12.5 mm is attained at a frequency of 9.5 GHz. Analogously, when considering slot lengths of 3 mm and 2 mm, a deterioration in the response of the reflection coefficient becomes evident. However, it is noteworthy that the optimal performance manifests at a slot length of 3.5 mm.

**Figure 6:**
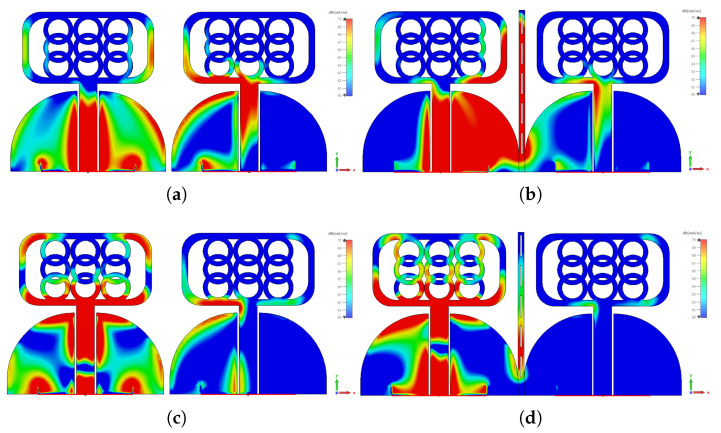

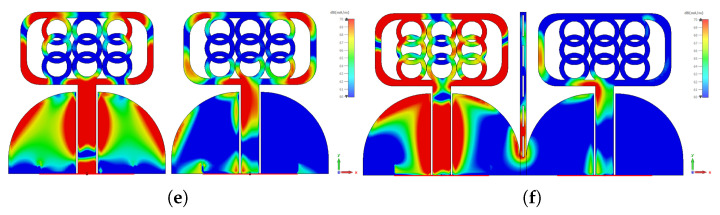
MIMO System Surface Currents with and without Isolating Structure: (**a**,**b**) 4GHz, (**c**,**d**) 6 GHz, (**e**,**f**) 10 GHz.

Figure 6 presents an investigation into the behavior of surface currents across different frequencies: 4 GHz, 6 GHz, and 10 GHz. The experiment focuses on a single antenna element emitting radiation while the remaining elements are terminated with matched impedance. The objective is to study the intriguing phenomenon of coupled fields and their impact on induced surface currents. Surprisingly, the non-radiating elements exhibit pronounced induced surface currents, both on the sides and facing sections. These currents arise due to the intricate interplay of coupled fields, creating a captivating scientific puzzle. Fortunately, a solution emerges in the form of a decoupling structure, which proves to be instrumental in mitigating these coupled effects. By introducing this structure, a remarkable transformation unfolds. Figure 6a showcases the current plot at 4 GHz, revealing a distinct alteration in the coupled currents. Now, these currents gracefully concentrate on the outer sections of the decoupling structure, indicating a resonant behavior at a lower frequency, as corroborated by Figure 6b. Similarly, Figure 6b demonstrates the current distribution at 6 GHz. With the decoupling structure in place, a captivating phenomenon unfolds: the currents are effectively suppressed. This suppression is attributed to the concerted efforts of both the middle and side sections of the decoupling structure, working synergistically to restore order amidst the coupled fields. Figure 6c presents the current distribution at the higher frequency of 10 GHz. Here, the decoupling structure demonstrates its full potential. Specifically, the middle section takes center stage, skillfully suppressing the induced currents. This empirical evidence is further bolstered by the supporting observations in Figure 4b, solidifying the importance of the decoupling structure in taming the currents at this elevated frequency range.

## 3. Results and Discussions

**Figure 7:**
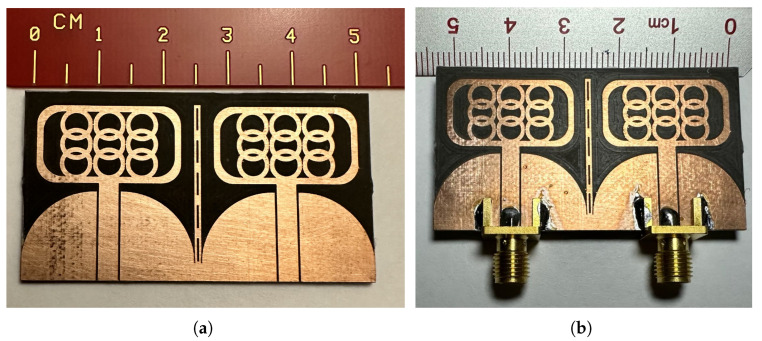
Fabricated Prototype (**a**) without Connectors and (**b**) with Connectors.

**Figure 8:**
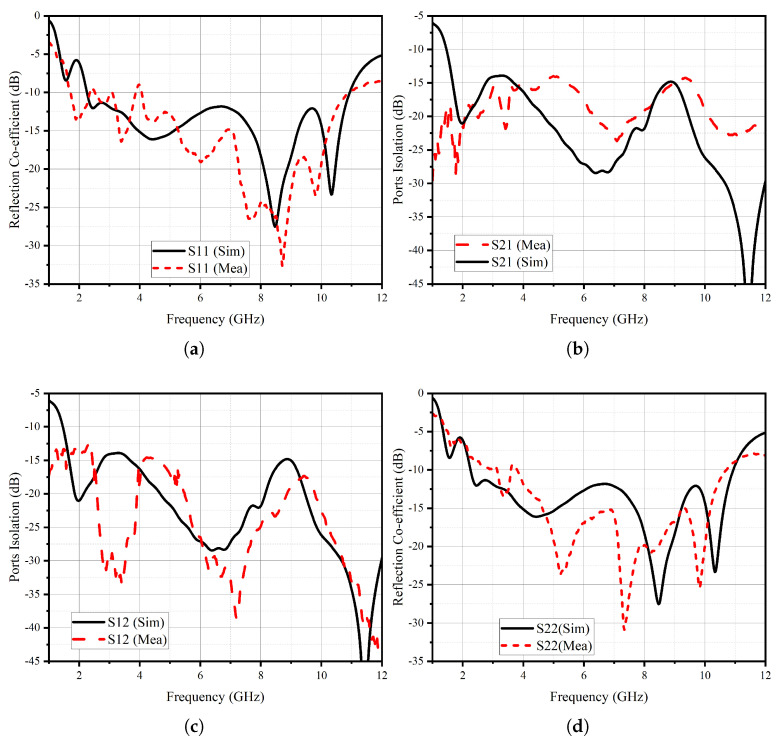
Simulated and Measured (**a**) S11, (**b**) S12, (**c**) S21, (**d**) S22.

As proposed, the UWB MIMO antenna system is fabricated and tested using our in-house facilities. The fabricated prototype is depicted in Figure 7, where a scale is included to verify the design dimensions. To compare the performance of the prototype with the simulated results, the s-parameters measured are shown in Figure 8. The solid black lines represent the results obtained from electromagnetic simulations using the finite difference time domain method, while the dashed red lines represent the measured data. It should be noted that the some moderate fluctuations observed in the measurements are primarily attributed to calibration drifts and the use of bent cables during the testing process.

### 3.1. S-Parameters

The antenna’s remarkable thinness and flexibility present a challenge in maintaining steady positioning during measurements. Nevertheless, Figure 8a,d illustrate a measured and simulated reflection coefficients. In regard to the MIMO antenna elements’ port isolation performance, Figure 8b,c display a comparison between simulations and measurements. The slight discrepancies observed can be attributed to calibration and substrate bend-related issues. Nevertheless, the achieved isolation performance is noteworthy, reaching a minimum of 16 dB, as depicted in the figures. While the measured isolation value lies slightly below at 14.5 dB, it still demonstrates commendable performance.

### 3.2. Radiation Patterns

**Figure 9:**
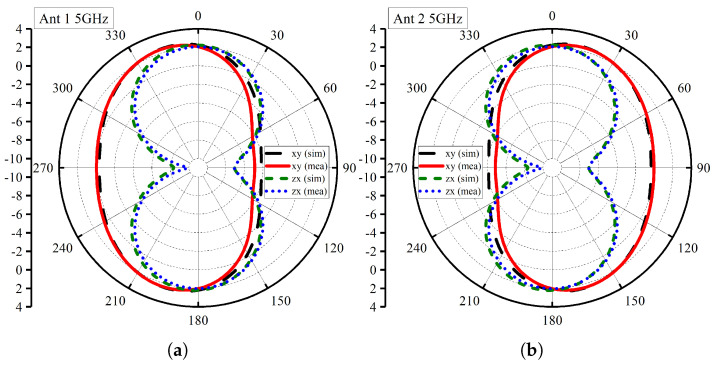

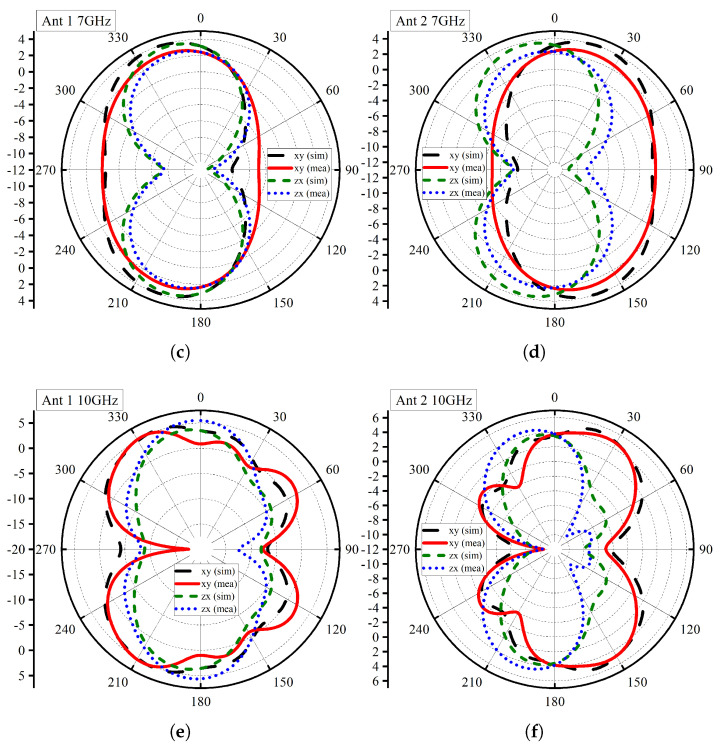
Radiation Patterns: (**a**) 5GHz Ant 1, (**b**) 5 GHz Ant 2, (**c**) 7 GHz Ant 1, (**d**) 7 GHz Ant 2, (**e**) 10 GHz Ant 1, (**f**) 10 GHz Ant 2.

Figure 9 illustrates the radiation characteristics of the proposed MIMO antenna, exhibiting its performance across multiple frequencies (5 GHz, 7 GHz, and 10 GHz). The analysis of port-1 individually reveals fascinating insights into the antenna’s behavior at different frequencies. At 5 GHz (Figure 9a), the antenna exhibits a highly desirable omnidirectional pattern in the xz-plane, ensuring uniform radiation coverage in all directions. Simultaneously, in the yz-plane, the pattern resembles that of a monopole, indicative of its efficient radiation in the vertical direction.

Advancing to 7 GHz (Figure 9b), the antenna maintains its excellent performance with a quasi-omnidirectional pattern in the xz-plane. This pattern ensures broad coverage in the horizontal plane, making it suitable for various communication applications. Additionally, the yz-plane continues to demonstrate a monopole-like pattern, showcasing its consistent performance across frequencies. At 10 GHz (Figure 9c), the antenna displays a quasi-omnidirectional pattern in both the xz-plane and yz-plane with minor fluctuations represented by slight ripples. Despite the higher frequency, the antenna maintains its stable and uniform radiation characteristics, making it well suited for modern high-frequency communication systems. It is crucial to highlight that the presented radiation pattern results exhibit remarkable agreement between simulation and measurement data, underscoring the reliability of the antenna’s performance analysis. This agreement validates the accuracy of the design and confirms the antenna’s consistent behavior across the specified frequencies.

## 4. MIMO Performance Parameters

**Figure 10:**
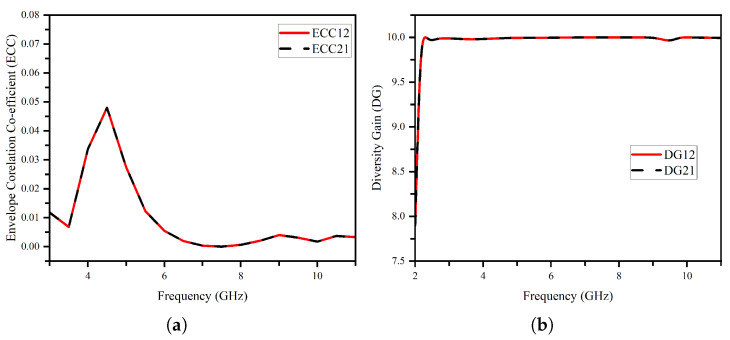
Time domain analysis configurations: (**a**) side-by-side and (**b**) face-to-face.

The MIMO performance parameters of the proposed UWB MIMO system is shown in Figure 10. ECC (envelope correlation coefficient) and diversity gain play crucial roles in assessing the antenna’s performance. The ECC is a measure of the correlation between the signals received or transmitted by different antenna elements. In the case of the studied UWB MIMO antenna, the obtained ECC values are exceptionally low, being less than 0.005 as shown in Figure 10, which indicates a minimal correlation between the two radiating elements. This low ECC value signifies improved spatial diversity and reduced interference, leading to enhanced system performance. Additionally, the diversity gain, calculated as the ratio of the received signal-to-noise ratio (SNR) with multiple antennas to the SNR with a single antenna, is reported to be 9.998 and above across the entire bandwidth. This substantial diversity gain further confirms the antenna’s ability to mitigate fading and improve the overall system reliability and capacity. The ECC and DG are derived through equations below.

(1) $$ECC=\frac{|{\;\int \int \;}_{4\pi }\left(\vec{B_{i}}\left(\theta ,\phi \right)\right)\times \left(\vec{B_{j}}\left(\theta ,\phi \right)\right){\;d\Omega |}^{2}}{{\;\int \int \;}_{4\pi }|\left(\vec{B_{i}}\left(\theta ,\phi \right)\right){|}^{2}\;d\Omega {\;\int \int \;}_{4\pi }{\left|\left(\vec{B_{j}}\left(\theta ,\phi \right)\right)\right|}^{2}\;d\Omega }$$

where $\vec{B_{i}}\left(\theta ,\phi \right)$ denotes the 3D radiation pattern upon excitation of the *i*th antenna and $\vec{B_{j}}\left(\theta ,\phi \right)$ denotes the 3D radiation pattern upon excitation of the *j*th antenna. Ω is the solid angle.

(2) $$DG=10\sqrt{1-(ECC^{2})}$$

## 5. Time Domain Analysis

Time domain analysis of antennas is a fundamental approach used to understand their behavior and performance over time. Unlike frequency domain analysis, which deals with the response of antennas to different frequencies, time domain analysis focuses on the temporal characteristics of the antenna’s signals. By studying how electromagnetic waves propagate through an antenna system in the time domain, engineers and researchers gain valuable insights into its transient behavior, radiation patterns, and response to different stimuli. One of the key aspects of time domain analysis is examining the antennas transient response after an excitation or input signal is applied. This analysis helps in understanding how the antenna reacts during its startup phase, enabling engineers to assess its stability, settling time, and overall performance during initial signal propagation.

Time domain analysis is particularly useful in assessing pulse antennas, which are designed to transmit and receive short-duration signals, such as radar pulses. Furthermore, time domain analysis provides valuable information about the antenna’s radiation patterns over time. By observing how the radiated electromagnetic fields evolve with respect to time, engineers can gain insights into the antenna’s directivity, gain, and efficiency. This temporal understanding is critical in applications where antennas need to steer their radiation patterns or adapt their responses dynamically. Time domain analysis also allows the investigation of antenna properties related to impedance and reflections. Engineers can assess how the impedance of an antenna changes over time, which is essential in impedance matching for efficient power transfer.

Additionally, studying reflections in the time domain helps identify potential signal distortions and interference issues that might arise in real-world scenarios. Moreover, time domain analysis plays a crucial role in antenna performance evaluation under various environmental conditions. By simulating the antenna’s response to dynamic changes in the surroundings, such as movement or varying channel conditions, engineers can optimize antenna designs for robustness and reliability. This is particularly important in wireless communication systems, where the antenna’s ability to handle fading and multipath propagation can significantly impact overall performance. In general, time domain analysis of antennas provides a comprehensive understanding of their transient behavior, radiation patterns, impedance characteristics, and response to environmental changes. This analysis aids in the design, optimization, and evaluation of antennas for a wide range of applications, including wireless communication, radar systems, and other time-critical scenarios. By delving into the temporal domain, engineers can unlock the full potential of antennas and ensure their efficient and reliable operation in various real-world settings.

The fidelity factor of an antenna is a critical parameter used to evaluate the performance of an antenna system in wireless communication. Also known as the antenna fidelity factor (AFF), it provides insight into how efficiently an antenna can transmit and receive signals with minimal distortion, thereby maintaining the integrity and accuracy of the data being transmitted. The fidelity factor takes into account several factors that affect an antenna’s performance. One of the primary considerations is the ability of the antenna to accurately radiate the electromagnetic signals it receives from the transmitter. A high fidelity factor implies that the antenna can efficiently convert electrical signals into electromagnetic waves and propagate them into free space with minimal loss or alteration. On the receiving end, the antenna should be able to capture and convert incoming electromagnetic waves back into electrical signals with high precision. Antennas with high fidelity factors are crucial in applications where signal integrity is paramount. For instance, in wireless communication systems used for data transmission, such as WiFi, cellular networks, or satellite communications, maintaining the fidelity of the signal is essential for ensuring data reliability and minimizing errors. To achieve a high fidelity factor, antenna design plays a vital role. Engineers focus on optimizing various antenna parameters, such as radiation efficiency, directivity, gain, bandwidth, and impedance matching. These factors collectively influence the antenna’s ability to faithfully reproduce the transmitted or received signals. Additionally, the surrounding environment can impact the fidelity factor. Obstructions, reflections, and multipath effects can lead to signal distortion and reduced accuracy in both transmission and reception. Antenna placement and orientation are critical considerations in mitigating such effects and enhancing the overall fidelity factor of the system. In short, the fidelity factor of an antenna is a measure of its ability to accurately transmit and receive signals with minimal distortion. A high fidelity factor is essential for maintaining signal integrity and data accuracy in various wireless communication applications. Engineers strive to optimize antenna design and placement to achieve superior performance and ensure reliable communication in our increasingly interconnected world. The FF is derived using the equation below. The FF of the proposed UWB antenna is shown in Table 1. In a face-to-face configuration, the FF value is high, which shows less distortion in the transmitted signal.

(3) $$FF=\max\left[\frac{\int_{-\infty }^{\infty }S_{t}\left(t\right)S_{r}\left(t+\tau \right)d\tau }{\int_{-\infty }^{\infty }|S_{t}{\left(t\right)|}^{2}dt\int_{-\infty }^{\infty }{\left|S_{r}\left(t\right)\right|}^{2}dt}\right]$$

where $S_{t}\left(t\right)$ corresponds to the transmitted signal and $S_{r}\left(t\right)$ represents the received signal, and the group delay is denoted by τ.

**Figure 11:**
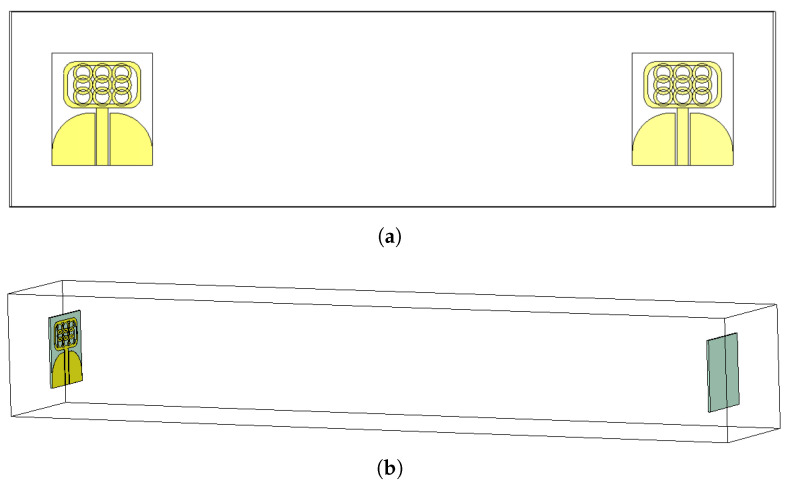
Time-domain analysis configurations: (**a**) side-by-side and (**b**) face-to-face.

**Figure 12:**
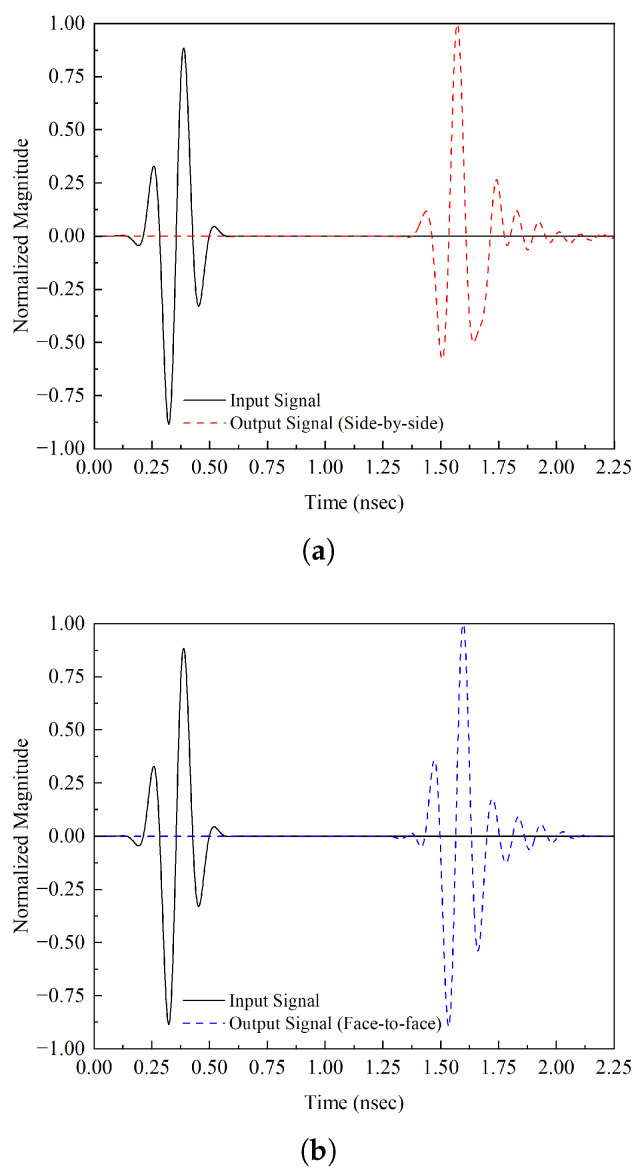
Time-domain analysis configurations: (**a**) side-by-side and (**b**) face-to-face.

**Figure 13:**
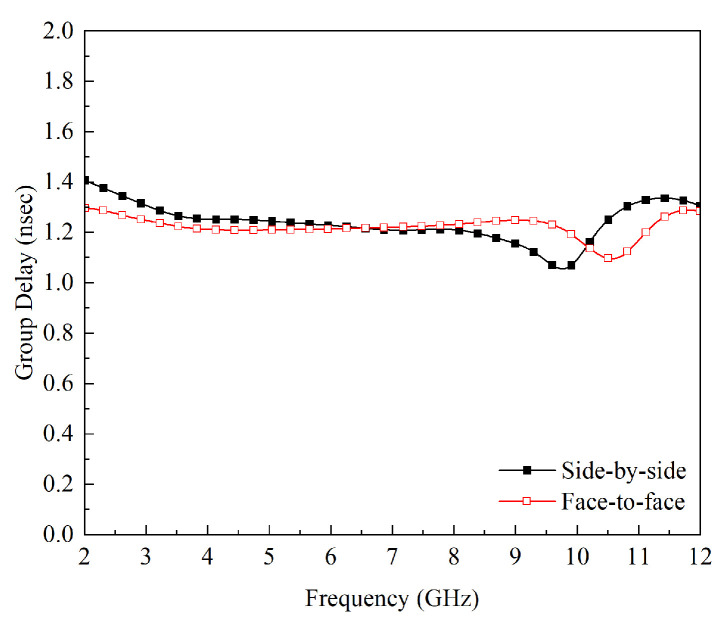
Group Delay of the Proposed UWB Antennas.

For UWB antennas, minimal distortion over the entire UWB range is desired. To find the dispersion characteristics and phase distortion of an antenna, the average delay between the center of the transient output and the input signal is measured; this phenomenon is known as group delay. Figure 11 demonstrates that both configurations have a peak value of 1.40 ns, as shown in Figure 12 indicating that less distortion will occur during the transmission of short pulses. The group delay is shown in Figure 13.

Table 2 presents a comparison between the proposed UWB MIMO antenna and the literature, focusing on key parameters, such as the number of elements in the system, frequency coverage, system size, ECC, and isolation levels between antenna elements. The findings reveal that the proposed UWB antenna demonstrates its suitability as a candidate for MIMO operations. By examining the table, it becomes evident that the proposed antenna outperforms the existing literature in terms of its compact size, wide frequency coverage, and remarkably low ECC values, indicating a minimal correlation between the antenna elements. Moreover, the isolation levels between the elements exhibit a significant improvement, further supporting the antenna’s potential for robust MIMO performance.

## 6. Conclusions

In this study, a two-port CPW-fed UWB MIMO antenna with a 52 × 26 mm size is presented. By incorporating an isolating structure (DGS), we achieved a significant improvement in isolation, up to 16 dB. The simulation and measurement results show excellent agreement, validating the accuracy of our design. The MIMO performance parameters, such as ECC and DG, are found to be in satisfactory limits. The time domain analysis of the proposed UWB antenna is also assessed and found to be in well-acceptable limits. The proposed MIMO antenna can be considered for its use in MIMO-based medical imaging systems which greatly rely on the efficiency of the antenna, and the increased values of isolation shall result in precise imaging. The proposed design presents a promising solution for enabling robust wireless connectivity, enabling seamless data transmission and supporting the growing demands of modern consumer electronics. By leveraging the advantages of UWB-MIMO technology, this antenna system can significantly improve the overall user experience, paving the way for advanced wireless applications in the realm of consumer electronics.

## Figures and Tables

**Table 1:** Fidelity factor of the proposed antenna.

Side by Side	Front by Front
90.31	−97.85

**Table 2:** Comparison of Proposed Antenna System with the Literature.

Ref	MIMO	Size	Bandwidth	Isolation	Gain	ECC
	Elements	(mm^2^)	(GHz)	(dB)		
10	2	26 × 20	3.1–10.6	>18	3.3	<0.16
11	2	30 × 18	4.5–15	>18	3	<0.12
12	2	60 × 60	3–12.8	>15	6.8	<13
13	2	33 × 48	12–13.7	>13	4.3	<0.08
14	2	40 × 68	3.2–10.6	>19	NA	<0.15
15	2	18 × 36	3–40	>18	6	<15
Prop.	2	52 × 26	2.3–11.5	>16	5.9	<0.012

## Data Availability

All the data are available in the study.
